# Curcumin reduces enteric isoprostane 8-iso-PGF2α and prostaglandin GF2α in specific pathogen-free Leghorn chickens challenged with *Eimeria maxima*

**DOI:** 10.1038/s41598-021-90679-5

**Published:** 2021-06-02

**Authors:** Victor M. Petrone-Garcia, Raquel Lopez-Arellano, Gabriela Rodríguez Patiño, Miriam Aide Castillo Rodríguez, Daniel Hernandez-Patlan, Bruno Solis-Cruz, Xochitl Hernandez-Velasco, Fernando Alba-Hurtado, Christine N. Vuong, Inkar Castellanos-Huerta, Guillermo Tellez-Isaias

**Affiliations:** 1 Programa de Doctorado en Ciencias de la Salud y Producción Animal, Universidad Nacional Autonoma de Mexico, Cuautitlan Izcalli, Estado de Mexico Mexico; 2 Laboratorio No 5: LEDEFAR, Unidad de Investigacion Multidisciplinaria, Facultad de Estudios Superiores Cuautitlan, Universidad Nacional Autonoma de Mexico, Cuautitlan Izcalli, Estado de Mexico Mexico; 3 Division de Ingenieria en Nanotecnologia, Universidad Politecnica del Valle de Mexico, Tultitlan, Estado de Mexico Mexico; 4 Departamento de Medicina y Zootecnia de Aves, Facultad de Medicina Veterinaria y Zootecnia, UNAM, Mexico, Mexico; 5 Departamento de Ciencias Biologicas, Facultad de Estudios Superiores Cuautitlan, Universidad Nacional Autonoma de Mexico, Cuautitlan Izcalli, Estado de Mexico Mexico; 6 Department of Poultry Science, University of Arkansas, Fayetteville, AR USA

**Keywords:** Biological techniques, Immunology, Microbiology, Gastroenterology

## Abstract

The purpose of this pilot study was to evaluate and determine the concentration of prostaglandin GF2α (PGF2α) and isoprostane 8‐iso‐PGF2α in plasma and intestine of specific pathogen-free (SPF) Leghorn chickens challenged with *Eimeria maxima*, with or without dietary supplementation of curcumin using solid‐phase microextraction and ultra‐performance liquid chromatography/tandem mass spectrometry. Eighty 1-day-old male SPF chickens were randomly allocated to one of four groups with four replicates (n = 5 chickens/replicate). Groups consisted of: (1) Control (no challenge), (2) Curcumin (no challenge), (3) *Eimeria maxima* (challenge), and (4) *Eimeria maxima* (challenge) + curcumin. At day 28 of age, all chickens in the challenge groups were orally gavaged with 40,000 sporulated *E. maxima* oocysts. No significant differences (*P* > 0.05) were observed in the groups regardless of the treatment or challenge with *E. maxima.* Enteric levels of both isoprostane 8‐iso‐PGF2α and PGF2α at 7 days and 9 days post-challenge were significantly increased (*P* < 0.01) compared to the non-challenge control chickens. Interestingly, the enteric levels of both isoprostane 8‐iso‐PGF2α and PGF2α at 7 days post-challenge were significantly reduced in chickens fed curcumin, compared to control chickens challenge with *E. maxima.* At 9 days post-challenge, only levels of isoprostane 8‐iso‐PGF2α in the enteric samples were significantly reduced in chickens challenged with *E. maxima* supplemented with curcumin, compared with *E. maxima* challenge chickens. No differences of isoprostane 8‐iso‐PGF2α or PGF2α were observed in plasma at both days of evaluation. Similarly, no significant differences were observed between the challenge control or chickens challenge with *E. maxima* and supplemented with curcumin at both times of evaluation. The results of this pilot study suggests that the antioxidant anti-inflammatory properties of curcumin reduced the oxidative damage and subsequent intestinal mucosal over-production of lipid oxidation products. Further studies to confirm and extend these results in broiler chickens are required.

## Introduction

Coccidiosis is a parasitic enteric disease of animals caused by coccidian protozoa from the Apicomplexa phylum. In a recent study, the global cost of coccidiosis in broiler chickens was estimated at ~ £10.36 billion^1^. In commercial poultry, coccidiosis has been controlled effectively with anticoccidial products, however, the extensive use of anticoccidial drugs has led to development of resistance against all these drugs^2^. To reduce the occurrence of resistance, the rotation of various anticoccidial drugs in single and shuttle programs is used^3^. Unfortunately, this has not solved the anticoccidial resistance problem. Live anticoccidial vaccines have been incorporated into rotation programs, resulting in an increased incidence of anticoccidial drug-sensitive *Eimeria* spp. field isolates, which may improve the efficacy of anticoccidial drugs^4^. Nevertheless, possible upcoming bans restricting the use of anticoccidials as feed additives, consumer concerns on residues, and increasing regulations have prompted the quest for alternative coccidiosis control strategies^5,6^. Although management and biosecurity measures could halt the introduction of *Eimeria* spp. to a farm, in practice, they do not suffice to prevent coccidiosis outbreaks.

Several phytogenics have been evaluated as feed additives in the poultry industry to protect feed from degradation and deterioration during storage, as well as for nutritional purposes^7^. However, it has been reported that these additives play an essential role in the prevention of several diseases in poultry due to their antioxidant, anti-inflammatory, antibacterial, antiviral, antifungal, and immunomodulatory properties^8–10^. Hence, in recent years, our laboratory has been evaluating curcumin as a feed additive to control *Salmonella* Enteritidis and necrotic enteritis in broiler chickens^11–13^. Curcumin is a bright yellow chemical and the principal curcuminoid of turmeric (*Curcuma longa*), a member of the ginger family (Zingiberaceae). For centuries, curcumin has been used as spice and food-coloring agent. In the poultry industry, curcumin has been used as anticoccidial, anti-inflammatory, immunomodulatory, antimicrobial, antioxidant and to promote growth performance^14–16^. Diets supplemented with of 1% of curcumin reduced intestinal lesion scores, oocyst per gram excretion (OPG) and improved weight gains during *E. maxima* infections and this anticoccidial activity was suggested to result from its antioxidant properties^17^. Other studies have been shown that curcumin inhibits induction of nitric oxide synthase in macrophages stimulated with endotoxin, as well as serum nitrogen dioxide and nitrate in *E. maxima*-infected chickens fed curcumin^18,19^.

*Eimeria* spp. have a remarkable and complex life cycle, including sexual and asexual reproduction with intracellular and extracellular phases^20–22^. Hence, during the disease, the gut-associated lymphoid tissues respond with a series of innate and acquired immune reactions against the parasite^23,24^. Several investigators have extensively studied and documented the immunopathology of cellular responses involving the secretion of pro-inflammatory cytokines to *Eimeria* infections in chickens^25–30^. However, little is known about the role of prostaglandins (PG) and isoprostanes (F_2_-Ips) as part of the innate response during clinical coccidiosis. Prostaglandins are a group of lipid compounds from the eicosanoid family implicated in inflammation, allergy, fever, and other immune responses that are generated from arachidonic acid by the action of cyclooxygenases (COXs) isoenzymes. Conversely, F_2_-Ips are PG-like complexes formed from free radical catalyzed oxidation of arachidonic acid, without the action of COXs. The measurement of F_2_-Ips, especially 8-epi-PGF_2α_, is recognized as a consistent biomarker of lipid peroxidation and is currently used as a sensitive index of oxidative stress in vivo.

The purpose of this pilot study was to evaluate and determine the concentration of prostaglandin GF2α (PGF2α) and isoprostane 8‐iso‐PGF2α in plasma and intestine of specific pathogen-free (SPF) Leghorn chickens challenged with *Eimeria maxima*, with or without dietary supplementation of curcumin, using solid‐phase microextraction and ultra‐performance liquid chromatography/tandem mass spectrometry.

## Results

The evaluation of body weight and body weight gain (in grams) of specific pathogen-free Leghorn chickens without or with *Eimeria maxima* challenge (7 days post-challenge) are summarized in Table 1. In the present study, challenge with 40,000 sporulated oocysts of *E. maxima* did not affect the body weight or body weight gain of SPF Leghorn chickens. No significant differences (*P* > 0.05) were observed in the groups regardless of the treatment or challenge with *E. maxima* (Table 1).

**Table 1:** Evaluation of body weight and body weight gain (in grams) of specific pathogen-free Leghorn chickens with or without *Eimeria maxima* challenge (7 days post-challenge).

Group	Body weight 21 days	Body weight 28 days	Body weight gain	Daily body weight gain
Control non-challenge	258.25 ± 9.28	275.55 ± 8.53	17.30 ± 2.11	2.47 ± 0.30
Curcumin non-challenge	254.95 ± 8.22	273.80 ± 8.14	18.85 ± 1.63	2.69 ± 0.23
*E. maxima* challenge	248.95 ± 6.91	266.30 ± 6.32	17.35 ± 2.07	2.48 ± 032
*E. maxima* + curcumin	251.40 ± 7.79	267.90 ± 8.51	16.50 ± 2.30	2.36 ± 0.29

Data expressed as mean ± standard error. *P* > 0.05.

**Figure 1:**
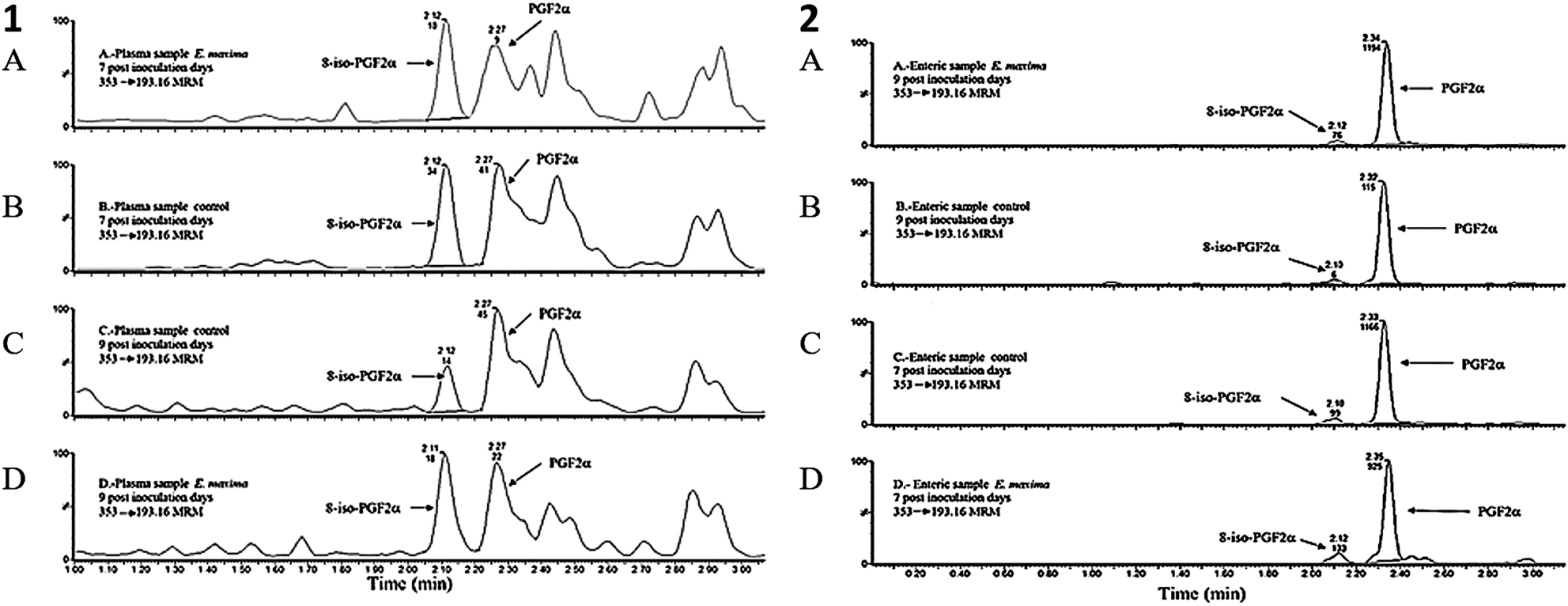
Chromatograms of 8-iso-PGF2α and PGF2α. (**1**) Obtained from plasma samples: A—sample of *E. maxima* 7 days post-inoculation chickens, B—sample of control of 7 days post-inoculation chickens, C—sample of control of 9 days post-inoculation chickens, and D—sample of *E. maxima* 9 days post-inoculation chickens. (**2**) Obtained from enteric (jejunum) samples: A—sample of *E. maxima* 9 days post-inoculation chickens, B—sample of control of 9 days post-inoculation chickens, C—sample of control of 7 days post-inoculation chickens, and D—sample of *E. maxima* 7 days post-inoculation chickens.

Table 2 presents the results of the evaluation of isoprostane 8‐iso‐PGF2α and PGF2α from jejunum and plasma in SPF chickens challenged with *E. maxima* at 7- and 9-days post-challenge. Enteric levels of both isoprostane 8‐iso‐PGF2α and PGF2α at 7 days and 9 days post-challenge were significantly increased (*P* < 0.01) compared to the non-challenge control chickens (Table 2; Fig. 1). Interestingly, the enteric levels of both isoprostane 8‐iso‐PGF2α and PGF2α at 7 days post-challenge were significantly reduced in chickens fed with curcumin compared to control chickens challenge with *E. maxima.* At 9 days post-challenge, only levels of isoprostane 8‐iso‐PGF2α in the enteric samples were significantly reduced in chickens challenged with *E. maxima* supplemented with curcumin, as compared with *E. maxima* challenge chickens. No differences of isoprostane 8‐iso‐PGF2α or PGF2α were observed in the plasma at both days of evaluation (Table 2; Fig. 1).

**Table 2:** Evaluation of isoprostane 8‐iso‐PGF2α and prostaglandin GF2α from enteric (jejunum) and plasma of specific pathogen-free Leghorn chickens at 7- and 9-days post-challenge.

Group	Prostaglandin GF2α		Isoprostane 8-iso-PGF2α	
	Enteric (pg/g)	Plasma (pg/mL)	Enteric (pg/g)	Plasma (pg/mL)
**7 days post-challenge**				
Control non-challenge	6934.47 ± 572.87^b^	107.92 ± 11.64	760.10 ± 75.56^b^	97.17 ± 8.96
Curcumin non-challenge	5843.27 ± 631.55^b^	117.29 ± 22.34	582.97 ± 70.68^b^	95.58 ± 15.27
*E. maxima* challenge	12,076.52 ± 770.55^a^	151.50 ± 17.87	1272.80 ± 81.97^a^	101.99 ± 17.87
*E. maxima* + curcumin	8,088.87 ± 698.27^b^	118.54 ± 9.89	864.93 ± 55.21^b^	97.97 ± 17.16
**9 days post-challenge**				
Control non-challenge	8984.66 ± 603.25^b^	162.05 ± 15.73	669.16 ± 81.47^b^	110.55 ± 9.22
Curcumin non-challenge	7606.78 ± 721.99^b^	121.19 ± 14.62	602.21 ± 79.93^b^	106.39 ± 19.53
*E. maxima* challenge	14,191.48 ± 750.61^a^	124.38 ± 11.82	1363.84 ± 89.12^a^	105.33 ± 8.83
*E. maxima* + curcumin	10,884.00 ± 740.07^a,b^	135.66 ± 22.59	834.82 ± 125.43^b^	104.83 ± 15.92

Data expressed as mean ± standard error.

^a,b^Different superscripts within columns and days indicate a significant difference at *P* < 0.01.

The results of the evaluation of *E. maxima* oocyst per gram in the feces of specific pathogen-free Leghorn chickens at 7- and 9-days post-challenge are summarized on Table 3. No significant differences were observed between the challenge control or chickens challenged with *E. maxima* and supplemented with curcumin at both times of evaluation (Table 3).

**Table 3:** *Eimeria maxima* oocyst per gram in the feces of specific pathogen-free Leghorn chickens at 7- and 9-days post-challenge.

Group	7 days post-challenge	9 days post-challenge
Non-challenge control	0 (0)^b^	0 (0)^b^
Non-challenge Curcumin	0 (0)^b^	0 (0)^b^
*E. maxima* challenge	24,240 (20,200)^a^	2750 (1700)^a^
*E. maxima* + Curcumin	22,593 (28,962.5)^a^	3023 (1650)^a^

Each value represents the mean (median).

^a,b^Values within groups columns with different superscripts differ significantly at *P* < 0.05.

## Discussion

Coccidiosis remains one of the most critical diseases in the poultry industry. Due to international regulations and consumer pressures, there is a need to develop alternatives for antibiotic growth promoters in animal and poultry feed. Phytogenics seem to be candidates of interest as alternatives to antibiotic growth promoters because they have been shown to control of *Eimeria* infections due to the association of coccidial infection with lipid peroxidation of the intestinal mucosa^31^. Other studies have confirmed the benefits of phytogenics in reducing gastrointestinal infections and increasing performance^32–34^. Moreover, several studies have confirmed the reduction *E. maxima* infection severity in broiler chickens due to curcumin’s antioxidant properties^17–19^.

In addition to the critical job of absorbing water and nutrients, enterocytes also play an essential role in the mucosal immune response, maintaining tolerance to beneficial microbiota, and identifying luminal pathogens. The invasion of *Eimeria* spp. in intestinal epithelial cells is a complex process that includes several events, beginning with the excystation of sporozoites after oral ingestion of the oocysts^35,36^. As intracellular parasites, attachment and invasion of the sporozoites to the host cell is recognized by Toll-like receptors 4 and 15, involved in pathogen recognition and activation of the mucosal inflammasome IL-1/IL-18 axis, which is responsible for recruiting and activating heterophils, natural killer cells, mast cells, macrophages, and increased production of transcription factor NF-κB^37–40^. Nevertheless, sporozoites have evolved a unique molecular system fueling motility and invasion of epithelial cells through gliding motility, allowing them to rapidly invade host cells and form an intracellular parasitophorous vacuole that protects them from the intracellular hostile environment^41–44^. Within this vacuole, these Apicomplexa parasites gain precious time to continue with their multifaceted life cycle. Each phase of the sexual, asexual, intracellular, or extracellular stages of this prehistoric and remarkable parasite are associated with severe local inflammation, autophagy, apoptosis, cellular death, hemorrhages, and necrosis in the intestinal mucosa^42–47^. Hence, coccidia infections are characterized by excessive tissue damage caused by the parasite infection and chronic inflammation of the host immune response elicited against the invaders. In chickens, macrophages are the primary sources of nitric oxide, superoxide, and hydrogen peroxide as essential mediators of both innate and acquired immunity, thus increasing during coccidia infections^48–52^. In the present study, chickens challenged with *E. maxima* presented with a significant increase (*P* < 0.01) in enteric PGF2α at 7- and 9-days post-challenge when compared with non-challenged chickens. However, the serum levels of PGF2α remained similar in both groups. Interestingly, chickens challenged with *E. maxima* and supplemented with curcumin showed a significant reduction of PGF2α levels at 7 days post challenge when compared with *E. maxima* control chickens. PGs are produced from arachidonic acid release from phospholipids in the cellular membrane by cyclooxygenases (COXs). They are fundamental in generating inflammatory responses against pathogens^53,54^. While they have a rapid response during the acute phases of the inflammatory response, there is crosstalk with cytokines to synergistically activate NF-κB factor and induce gene expression of pro-inflammatory cytokines and more COXs, mediating positive feedback loops and consequently, chronic inflammation^55,56^.

Since the cellular components that suffer immediate damage are the lipids and proteins of the cell membrane and mitochondrial membrane by lipid peroxidation, the whole-cell physiology is then compromised. One of the cellular mechanisms to revert oxidative stress is the production of several heat shock proteins that repair damage proteins and regulate apoptosis^57–59^. A noteworthy result observed in this study was the significant increase in isoprostane 8‐iso‐PGF2α in the jejunum of chickens challenged with *E. maxima* at 7- and 9-days post-challenge compared to the non-challenge control chickens. Furthermore, chickens in the group supplemented with curcumin showed a significant reduction in isoprostane 8‐iso‐PGF2α in the jejunum of chickens challenged with *E. maxima* at both days of evaluation post-challenge compared to the *E. maxima* challenge control chickens. Excessive generation of reactive oxygen species has been implicated in a variety of pathological events. However, lipid peroxidation is the primary marker of oxidative stress in many pathological conditions, so isoprostanes are reliable evaluation biomarkers evaluate^60,61^. In contrast, F2-isoprostanes (8-Iso-PGF2α) have harmful and potent bioactivities, including vasoconstriction, platelet aggregation, and cardiac hypertrophy^62–65^. As far as we know, this is the first report of detection of 8-Iso-PGF2α following a challenge of *E. maxima* in the jejunum, as well as demonstrating the protective antioxidant properties of curcumin reducing the enteric levels of 8-Iso-PGF2α, despite plasma levels of 8-Iso-PGF2α remaining similar in all groups, regardless of the challenge with *E. maxima*. It is known that in humans, the plasma half-life of 8-Iso-PGF2α is one minute at the distribution stage and the removal stage half-life is four minutes^66^. Hence, the half-life in chicken plasma may also be short, which may be why we were not able to detect it. However, pharmacokinetic and metabolic studies evaluating earlier points as well as daily oocyst count are required to confirm and extend these results.

In summary, in the present study, SPF Leghorn chickens challenged with *E. maxima* showed an inflammatory response associated with a significant increase at 7 days and 9 days post challenge in enteric PGF2α. These changes were related to a significant increase of enteric 8-Iso-PGF2α and oocyst excretion at both days of evaluation, suggesting that the active disease phase was accompanied by inflammation and oxidative stress within the intestinal layer. Nevertheless, dietary supplementation of curcumin reduced the levels of PGF2α and 8-Iso-PGF2α at 7 days post challenge, and 8-Iso-PGF2α at 9 days post challenge compared with *E. maxima* challenged control chickens. Since polyunsaturated fatty acids and cholesterol are the principal targets of oxidative stress, lipid peroxidation end products, such as 8-Iso-PGF2α, are also a part of the pathogenesis of inflammation-related changes caused by *E. maxima*, confirming the role of 8-Iso-PGF2α as a sensitive biomarker of oxidative stress in chickens. The results of this pilot study suggest that the antioxidant and anti-inflammatory properties of curcumin are able to reduce oxidative damage and subsequently intestinal mucosal over-production of lipid oxidation products. Further studies to confirm and extend these results in broiler chickens are required.

## Methods

### Challenge strain

*Eimeria maxima* M6 oocysts were provided by Dr. John. R. Barta, University of Guelph, Canada. The methods for detecting and recovering oocysts from challenged chickens, oocyst sporulation, and the preparation of infective doses were conducted as described previously^67,68^.

### Starter diet

A control basal non-supplemented diet and a basal diet supplemented with 2% curcumin were used in this experiment (Table 4). Starter feed used in this experiment was formulated to approximate the nutritional requirements for Leghorn chickens as recommended by the National Research Council^69^ and adjusted to Hy-Line Management Guide, W36 Commercial Layers recommendations^70^. No antibiotics, coccidiostats, or enzymes were added to the feed.

**Table 4:** Ingredient composition (kg) and nutrient content of feed supplied to the experimental SPF chickens.

Ingredients	Pre-starter (0–3 weeks)
Yellow corn 7.1%	622.62
Soybean meal 46.5%	323
Limestone 38% Ca	18
Phosphate 21/27%	12
Vegetable oil	10
NaCl (refined salt)	4
Vitamin premix^a^	1.4
Mineral premix^b^	1.1
DL-Methionine 99%^c^	3.700
Liquid l-lysine 50%^d^	3.500
l-Threonine^e^	0.640
6-Phytase^f^	0.040
**Nutrients**^70^	
Weight	1.0
Dry matter (%)	88.300
Crude protein (%)	20.000
Metabolizable energy (Mcal kg^−1^)	3.087
Choline (mg kg^−1^)	2.000
Arginine (%)	1.210
Linoleic acid (%)	1.200
Total lysine (%)	1.150
Total calcium (%)	1.050
Methionine + cystine (%)	0.830
Valine (%)	0.830
Threonine total (%)	0.820
Isoleucine (%)	0.790
Methionine total (%)	0.510
Phosphorus available (%)	0.480
Phosphorus digestible (%)	0.440
Total tryptophan (%)	0.210
Total chlorine (%)	0.180
Total sodium (%)	0.180

^a^Vitamin premix supplied per kg of diet: Retinol, 6 mg; cholecalciferol, 150 µg; dl-α-tocopherol, 67.5 mg; menadione, 9 mg; thiamine, 3 mg; riboflavin, 12 mg; pantothenic acid, 18 mg; niacin, 60 mg; pyridoxine, 5 mg; folic acid, 2 mg; biotin, 0.3 mg; cyanocobalamin, 0.4 mg.

^b^Mineral premix supplied per kg of diet: Mn, 120 mg; Zn, 100 mg; Fe, 120 mg; copper, 10 to 15 mg; iodine, 0.7 mg; selenium, 0.2 mg; and cobalt, 0.2 mg.

^c^MetAMINO® (Evonik, Essen, Germany).

^d^Liquid l-lysine 50% (ADM, Chicago, IL, USA).

^e^ThreAMINO® (Evonik, Essen, Germany).

^f^Axtra PHY TPT 10,000® (Dupont Industrial Biosciences, Marlborough, UK).

### Animal source and experimental design

Eighty one-day-old male specific pathogens-free (SPF) Leghorn chickens (ALPES® Tehuacan, Puebla, Mexico) were randomly allocated to one of four groups with four replicates per group (n = 5 chickens/replicate). Chickens were placed in battery cages with a controlled age-appropriate environment at the diagnostic laboratory of the Avian Medicine Department of the Faculty of Veterinary Medicine and Zootechnics (FMVZ) at the National Autonomous University of Mexico (UNAM). Groups consisted of: (1) Control (no challenge), (2) Curcumin (no challenge), (3) *Eimeria maxima* (challenge), and (4) *Eimeria maxima* (challenge) + curcumin. Chickens were provided with ad libitum access to water. At day 28 of age, all chickens in the challenge groups were orally gavaged with 40,000 sporulated *E. maxima* oocysts in a volume of 1 mL of sterile phosphate-buffered saline solution (PBS). The dose used in the present study did not cause clinical coccidiosis in SPF Leghorn chickens. The dose was selected based on a previous trial conducted to determine a challenge dose causing sub-clinical coccidiosis as described previously^13^. Negative control chickens were sham inoculated with 1 mL of PBS. Seven days after challenge, all chickens were bled, and half of them were euthanized to collect the second half of the jejunum to determine plasma and enteric concentrations of isoprostane 8‐iso‐PGF2α and PGF2α. At 9 days post-challenge, remaining chickens from all groups were bled and jejunum was collected to perform the evaluations. Oocysts per gram (OPG) of feces were evaluated at 7- and 9-days post-challenge.

### The standards for 8‐iso‐PGF2α and 8‐iso‐PGF2α‐d4

The standards for 8‐iso‐PGF2α and 8‐iso‐PGF2α‐d4 (internal standard) were purchased from Cayman Chemicals (Ann Arbor, MI), while the standard for PGF2α was obtained from Sigma-Aldrich (St Louis, MO). Acetonitrile and methanol (HPLC grade) were purchased from JT Baker. Milli‐Q water (Millipore system) was used throughout the experiments. Formic acid (FA: 95%, reactive grade) and isopropanol (LC/MS grade) were purchased from Sigma-Aldrich (St Louis, MO). Ammonium hydroxide (NH_4_OH, reactive grade, 29.60%) and potassium hydroxide (KOH) were purchased from JT Baker. For solid‐phase microextraction (micro‐SPE), 96‐well Oasis® MAX μElution cartridges containing a water‐wettable reversed‐phase strong ammonium exchange mixed‐mode polymer, which is selective for acids and stable in organic eluents, were used. A Positive Pressure‐96 processor purchased from Waters was also used. Figure 1 shows the chromatograms of standards.

### Procedure for the extraction of 8-iso-PGF2α and PGF2α in chicken plasma

Extraction of 8-iso-PGF2α and PGF2α were determined as previously described^71^. An aliquot of 500 µL chicken plasma was transferred to 2 mL vials, followed by the addition of 100 µL of 4 ng/mL 8‐iso‐PGF2α‐d4 as an internal standard and 500 µL of hydrolysis solution (KOH, 15%) to release 8-iso-PGF2α-esterified. The vials were mixed and incubated in an ultrasonic bath for 30 min at 40 °C. Subsequently, the vials were cooled to room temperature and 225 µL of 6 M formic acid (FA) was added, mixed, and centrifuged at 15,000 rpm for 10 min at 4 °C. Solid‐phase microextraction using a 96‐well Oasis® MAX μElution plate conditioned with 500 μL of methanol and 500 μL of 20 mM FA was used. Finally, the cartridges were loaded with 350 μL of plasma and washed with 350 μL of 2% NH_4_OH. Samples were then eluted with 50 μL of a mixture of 5% FA in acetonitrile and isopropanol (40:60) and diluted with 150 μL of Milli‐Q water. Samples were analyzed (30 μL) using ultra‐performance liquid chromatography/tandem mass spectrometry (UPLC/MS/MS).

### Procedure for the extraction of 8-iso-PGF2α and PGF2α in chicken intestine

For the extraction of 8-iso‐PGF2α and PGF2α, 0.1 g of homogenized second half of the jejunum (Meckel’s diverticulum to cecal tonsils) were transferred to 2 mL vials, followed by the addition of 100 µL of 4 ng/mL 8‐iso‐PGF2α‐d4 as the internal standard and 1.5 mL of chloroform: methanol (80:20) mixture. The vials were mixed 30 s by vortex and 15 min in an ultrasonic bath. Samples were then centrifuged at 15,000 rpm for 20 min. The supernatant was evaporated and 500 µL of methanol and 500 µL of hydrolysis solution (KOH 15%) were added, mixed, and incubated in an ultrasonic bath for 30 min at 40 °C. Subsequently, the vials were cooled to room temperature and 225 µL of 6 M formic acid (FA) and 50 µL of 88% FA were added, mixed, and centrifuged at 15,000 rpm for 10 min at 4 °C. Solid‐phase microextraction and analysis of samples were performed in the same way as for the determination of 8-iso-PGF2α and PGF2α in chicken plasma using a 96‐well Oasis® MAX μElution plate conditioned with 500 μL of methanol and 500 μL of 20 mM FA. Finally, the cartridges were loaded with 350 μL of jejunum sample and washed with 350 μL of 2% NH_4_OH. Samples were then eluted with 50 μL of a mixture of 5% FA in acetonitrile and isopropanol (40:60) and diluted with 150 μL of Milli‐Q water. The sample (30 μL) was injected into a UPLC-MS/MS system for analysis, under the chromatographic and mass spectrometric conditions described previously by Rodriguez Patiño et al.^71^.

### Ethics

This study was carried out in accordance with the guidelines for the management of chickens as recommended by the Internal Committee for Care and Use of Experimental Animals (CICUAE, from its abbreviation in Spanish) of the National Autonomous University of Mexico (UNAM), Ethical approval code CICUAE: C20_06, and the study is in compliance with the ARRIVE guidelines where animals are involved.

### Quantification of oocysts

The quantification of OPG from feces was performed at 7- and 9-days post-challenge by using the McMaster technique as previously described^67^.

### Data and statistical analysis

PGF2α and 8-iso-PGF2α data are presented as means with standard deviation (S.D.). The number of samples per variable group was 20, implying a normal distribution (Shapiro–Wilk test), and the homoscedasticity was verified (Levene's test). Accordingly, the parametric test of analysis of variance (ANOVA) was performed, and the differences between the means were evaluated using Tukey’s honestly significant difference (HSD) test, and the *P* value was established with an alpha level of *P* < 0.01. OPG data are presented as means with median. The number of samples per variable group was 20; however, the hypotheses of normal distribution (Shapiro–Wilk test) and homoscedasticity (Levene’s test) were not confirmed. Consequently, non-parametric tests of non-parametric tests of the two-tailed Kruskal–Wallis was applied and subsequently the Mann–Whitney’s U test to compare between pairs of groups was applied with an alpha level *P* < 0.05^72^.
